# Green tea consumption is associated with reduced incident CHD and improved CHD-related biomarkers in the Dongfeng-Tongji cohort

**DOI:** 10.1038/srep24353

**Published:** 2016-04-13

**Authors:** Chong Tian, Qiao Huang, Liangle Yang, Sébastien Légaré, Francesca Angileri, Handong Yang, Xiulou Li, Xinwen Min, Ce Zhang, Chengwei Xu, Jing Yuan, Xiaoping Miao, Mei-an He, Tangchun Wu, Xiaomin Zhang

**Affiliations:** 1 School of Nursing, Tongji Medical College, Huazhong University of Science and Technology, Wuhan, China; 2 Department of Occupational and Environmental Health and Ministry of Education Key Lab for Environment and Health, School of Public Health, Tongji Medical College, Huazhong University of Science and Technology, Wuhan, China; 3 Dongfeng Central Hospital, Dongfeng Motor Corporation and Hubei University of Medicine, Shiyan, China; 4 Department of Epidemiology and Statistics, School of Public Health, Tongji Medical College, Huazhong University of Science and Technology, 13 Hangkong Rd, Wuhan, China

## Abstract

Prospective studies on the association of green tea with risk of coronary heart disease (CHD) incidence were scarce. This study examined whether green tea can reduce CHD incidence and have a beneficial effect on CHD-related risk markers in middle-aged and older Chinese population. We included 19 471 participants who were free of CHD, stroke or cancer at baseline from September 2008 to June 2010, and were followed until October 2013. Cox proportional hazard models were used to examine the hazard ratios (HR) of CHD incidence in relation to green tea consumption. Linear regression models were used to evaluate the effect of green tea on 5-year changes of CHD-related biomarkers. Compared with non-green tea consumers, the multivariable-adjusted HR for CHD was 0.89 (95% CI, 0.81-0.98) in green tea consumers. Particularly, the reduced risk of CHD incidence with green tea consumption was more evident among participants who were male, more than 60 years old, overweight, or with diabetes mellitus. In addition, green tea consumption improved multiple CHD-related risk markers including total cholesterol, HDL-cholesterol, triglycerides, mean platelet volume, and uric acid. In conclusion, green tea consumption was associated with a reduced risk of CHD incidence in the middle-aged and older Chinese populations, and the association might be partly due to altered CHD-related biomarkers.

Cardiovascular disease (CVD) has become the leading cause of death and continues to exert a heavy burden in China. The number of patients with CVD increased to 290 million in 2014 with one out five Chinese adults suffering from the disease^1^. Coronary heart disease (CHD) accounts for the greatest proportion of CVD^2^, and the prevalence of Chinese people with CHD is increasing substantially due to ageing, imbalanced diets, unhealthy behaviors and the raising standard of living^3^. Thus, early prevention and control of CHD has become an extremely important public health concern.

Tea, a beverage made from leaves of *Camellia sinensis*, is the second most consumed beverage worldwide, only close to plain water^4^. Tea was generally categorized into black, green and oolong tea according to the manufacturing process. Black tea, which covers about 78% of the total tea production, is usually consumed in the West; whereas green tea, which covers 20%, is consumed primarily in Japan, China and other East Asian countries^5^. A recent meta-analysis of prospective studies based mainly on evidence from black tea or unspecified tea showed that tea consumption was associated with a reduced risk of CHD, and found that the associations between tea consumption and cardiovascular outcomes differ according to sex, ethnicity, and the type of tea consumed^6^. On the contrary, data on the association between green tea and CHD was limited and inconclusive. Several cohort studies have suggested an inverse association between green tea and CVD mortality^7^,^8^,^9^,^10^. Although dose-response relationship between green tea and risk of CHD was reported in case-control and cross-sectional studies^11^,^12^,^13^, only two cohort studies have examined the impact of green tea consumption on risk of CHD incidence and no significant association was observed^11^,^14^. On the other hand, favorable changes in CHD-related biomarkers, such as blood pressure, LDL cholesterol and glucose levels, were reported with green tea in randomized controlled trials^15^,^16^,^17^,^18^, which suggested a possible protective effect of green tea against CHD. However, most existing studies have only focused on one aspect: either green tea versus CHD, or green tea versus biomarkers; seldom studies have so far examined both of them on a same population.

Because of the high consumption of green tea in China and relatively high rate of CHD incidence and mortality, exploring the health effects of green tea on CHD might provide clues to resolve an important public health issue. Therefore, we aimed to 1) verify the relationship between green tea and CHD incidence in the Dongfeng-Tongji cohort; 2) examine whether the association between green tea and CHD was modified by differences in population characteristics and disease status; and 3) evaluate the effect of green tea on changes of multiple CHD-related biomarkers.

## Results

### Baseline characteristics of study population

As shown in Table 1, in 19 471 eligible participants, a total of 7021 were green tea consumers (36.06%). Men and participants with higher education level were more likely to consume green tea. The mean age of green tea consumers was higher than that of non-green tea consumers, and the age distribution was different. Higher rate of smoking and alcohol drinking, two of the most common and inveterate habits linked with poor health in China, were also observed in green tea consumers. Meanwhile, green tea consumers more often tended to be physically inactive, with higher BMI and uric acid level, and with hyperlipidemia. However, they exhibited lower levels of total cholesterol and LDL-C.

### Green tea consumption reduced incident CHD risk

Table 2 presents the 5-year cumulative CHD incidence rate of green tea consumers compared to non-green tea consumers, along with adjusted HR of incident CHD. Overall, green tea consumers had a reduced CHD incidence of 9.80% compared to 11.06% for non-green tea consumers (*P* = 0.006). In crude models, green tea consumption was associated with lower risk of CHD incidence (HR = 0.88, 95% CI = 0.80–0.96). After adjustment for age, sex, BMI, education, smoking status, alcohol drinking status, physical activity, hypertension status, hyperlipidemia status, diabetes status and family history of CHD at baseline, these associations became more obvious (HR = 0.85, 95% CI = 0.77–0.94). Additional adjustment for four other common beverages including coffee, black tea, juice and cola, the effect was slightly attenuated, but still remained statistically significant and corresponded to an 11% reduced risk of CHD incidence (HR = 0.89, 95% CI = 0.81–0.98).

We subsequently conducted stratified analyses by major characteristics of the study population. Substantial reduction of CHD incidence with green tea consumption was only seen in participants who were male, older than 60 years, overweight (BMI ≥ 25) or with diabetes mellitus. No significant interactions between green tea consumption and these characteristics on risk of CHD were observed.

### Green tea consumption improved CHD-related biomarkers

As shown in Table 3, the mean levels of total cholesterol, LDL-C, and MPV were significantly decreased, while HDL-C and uric acid were significantly elevated at 5-year follow-up compared to baseline in both green tea consumers and non-green tea consumers (all *P* < 0.001). On the contrary, significantly increased levels of triglycerides (*P* < 0.001) and fast glucose (*P* = 0.028) were only observed in non-green tea consumers. Moreover, after adjustment for age, sex, BMI, education, smoking status, alcohol drinking status, physical activity and the baseline level of each biomarker, green tea consumers had greater reductions in total cholesterol and MPV, greater increase in HDL-C, and smaller increase in triglycerides and uric acid than did non-green tea consumers over time (all *P* < 0.05). Overall, each tested biomarker improved more or worsened less over 5 years for green tea consumers.

## Discussion

The present study demonstrated that green tea consumption was independently related to an 11% reduction of CHD incidence, notwithstanding green tea consumers appear to have an unfavorable risk profile. Our findings add a considerable body of knowledge to previous studies. Some prior case-control or cross-sectional studies conducted in Japan generated inconsistent results regarding green tea and angiographically proven CHD. Several of those studies showed a protective relationship between green tea intake and CHD or coronary atherosclerosis^11^,^13^, whereas Hirano *et al.* did not find any inverse association of green tea intake with CHD^19^. Evidence in Chinese population was also limited. In a case-control study, Wang et al. found that green tea intake was associated with a reduced risk of CHD among Chinese male patients, but not in female patients^12^. More recently, Pang et al. also reported a dose-response relationship between green tea consumption and the risk of CHD in male patients^20^. Although insightful, those studies were case-control or cross-sectional, and may yield recall or reverse causality biases. Our prospective study is the first to agree with the majority of previous case-control or cross-sectional studies on the positive relationship between green tea and risk of CHD. Until now, no prospective study had shown evidence that green tea consumption is preventive of CHD incidence, as they generally indicated beneficial effects on CVD mortality only^7^,^8^,^9^,^10^. However, participants who consumed green tea in these cohort studies^7^,^8^,^9^,^10^ more often tended to have unfavorable risk profiles such as being older, current smoker, current alcohol drinker, having a history of hypertension or diabetes, or less likely to walk, which was consistent with the characteristics of our green tea consumers.

Several reasons could explain the disparity between our findings and those from previous prospective studies. Sano et al. found that green tea intake was not a predictor of CHD events after 4.9 ± 0.1 years follow-up, partly because of the small number of CHD events (n = 38)^11^. In another large prospective study conducted in a Japanese population, inverse associations of green tea consumption were only observed with the incidences of CVD and strokes, but not with CHD^14^. In the study from Japan, the mean age of participants was about 53 years at baseline and the average follow-up duration was 13 years; while in our study, the participants had a mean age of 62.8 years at baseline and they were only followed for about 5 years. Meanwhile, in that study, only MI and cardiac death was defined as outcomes. A total of 910 CHD events in 82 369 subjects (1.10%) were documented during a 13-year follow-up. In contrast, our study included SA, UA, MI, unspecified CHD and cardiac death as endpoints, and a total of 2065 cases were documented from 19 471 subjects (10.61%). Therefore, the low incidence rate and insufficient statistical power might be a possible reason for not detecting a significant effect of green tea on CHD incidence in that study. In addition, the disparate results might also be due to inherent differences in population characteristics such as age distribution, different definitions of end points, the processing of the tea, and sample size.

Interestingly, the above-mentioned association between green tea consumption and reduced risk of CHD incidence appeared to be more pronounced in individuals who were male, more than 60 years old, overweight or with diabetes, but no interaction noted. Consistent with the observations of our subgroups, results in all subgroups defined by traditional CVD risk factors from a former study also showed no interactions between green tea consumption and CVD risk factors on CVD mortality^10^. We cannot explain the underlying mechanism for this observation, but similar results in males were reported from several previous studies^12^,^13^. It is believed that estrogen may reduce the risk of CHD, while Wu et al. suggested that green tea consumption could decrease circulating estrogen levels^21^. So, we speculate that in female participants the protective association with green tea would be masked by this uncontrolled confounding variable. As green tea has been reported to generate weight loss, anti-aging, anti-diabetic^22^ and anti-androgen^23^ effects, individuals who were more than 60 years old, overweight or suffering from diabetes tended to consume more green tea to counteract these problems, thus, a stronger protective effect of green tea against CHD was observed in these high-risk populations. However, we could not directly compare our results with previous studies because no similar findings were reported.

Although the biological mechanisms underlying the protective effects of green tea on CHD remain unclear, most of randomized controlled trials have demonstrated beneficial effects of green tea on CVD risk profiles^17^,^24^. We therefore simultaneously explored the changes of CHD-related biomarkers at 5-year follow-up compared to baseline. We observed that green tea consumers had a greater reduction in total cholesterol, smaller increase in triglycerides, and a greater increase in HDL-C, which was in accordance with previous human and animal studies^25^. Similarly, green tea consumers also exhibited greater decrease in MPV, an indicator of platelet activation. A high MPV value represents an increase in platelet aggregation, thromboxane synthesis, and adhesion molecules expression^26^,^27^. An elevated MPV has also been associated with an increased risk of CHD^28^,^29^. Our results supported previous evidence that green tea had anti-platelet and anti-thrombotic activity^30^,^31^. In addition, despite higher baseline levels of uric acid, another risk marker of incident CHD^32^, we also found a smaller increase of uric acid in green tea consumers, in agreement with clinical trials^33^,^34^. In general, the above favorable changes in CHD-related biomarkers could be partially responsible for the protective effect of green tea on CHD incidence, whereas other mechanisms including radical scavenging, antioxidant bioactivity, and improvement of endothelial function still need further investigation.

There are several limitations in the present study. First, green tea consumption was based on self-reporting by the participants and only documented as green tea consumers or non-green tea consumers. The amount, frequency, duration, concentration of green tea consumption and other details were not included in the questionnaire. Thus we cannot perform a dose-response analysis to quantitatively elucidate the relationship between tea consumption and incident CHD or changes in CHD-related biomarkers. Second, we considered tea consumption as a lifelong habit, and did not perform a second check to determine status of consumption. Third, the study was carried out in middle-aged and older Chinese who were free of CVD and cancer; therefore our results may not be generalized to populations of all ages, different health conditions, or other ethnicities. Fourth, we did not adjust for multiple testing because the repeated measurements of biomarkers at baseline and follow up were highly correlated in our study. Over adjustment for multiple comparisons may increase the type II error, which reduces the power to detect significant differences. Finally, although a wide range of potential confounding factors were included in our analysis, we could not rule out the possibility of residual and unmeasured confounders.

This study showed that green tea consumption was associated with a reduced risk of CHD incidence, especially among individuals who were male, more than 60 years old, overweight, or suffering from diabetes. Substantial improvement in the changes of lipid profiles, MPV and uric acid might partly explain the potential beneficial effect of green tea on CHD incidence. Our results provided interesting and important evidence that are relevant to the field of CHD prevention. However, these findings need to be verified in future intervention studies with large sample sizes.

## Methods

### Study population

Data was collected from the Dongfeng-Tongji cohort, and the details of the cohort were described in a previous report^35^. Briefly, a total of 27 009 retired employees of the Dongfeng Motor Corporation (DMC) completed baseline questionnaire, medical examinations and provided fasting blood samples between September 2008 and June 2010. Five years later, the participants were invited to the follow-up survey via telephone. In total, 25978 individuals (96.2% of those at baseline) completed the follow-up until October 2013. At the follow-up investigation, participants repeated the questionnaire interview, physical examinations, and blood collection as those during the baseline survey. Of these 25978 individuals, we excluded participants with cancer, CHD or stroke at baseline (*n* = 5865), who had missing data on beverage consumption or other covariates (*n* = 642). Thus, 19 471 participants with a mean age of 62.8 ± 7.7 years were eligible, of which 8585 were males (65.8 ± 6.7 years) and 10 886 were females (60.5 ± 7.7 years). Among them, 2065 new onset CHD cases were documented. Informed consent was obtained from every participant. The protocol was approved by the Ethics Committee of Dongfeng General Hospital, DMC and the Medical Ethics Committee of the School of Public Health, Tongji Medical College, Huazhong University of Science and Technology, and the study was subsequently carried out in accordance with the approved guidelines.

### Baseline assessment

Trained interviewers used a structured questionnaire to collect baseline information including socio-demographic characteristics (age, sex, education, and marital status), lifestyle habits such as smoking status (current, former, never), alcohol drinking status (current, former, never), physical activity, occupational history, environmental exposures, and family and medical histories in face-to-face interviews. Beverage consumption was assessed by asking: “Did you usually consume beverages including plain water, black tea, green tea, coffee, cola and juice during the last year?” and participants were asked to select one or two most often consumed beverages. Green tea consumers were defined as participant who selected green tea as one of the most often consumed beverages. Participants who were smoking at least one cigarette per day for more than half a year were defined as current smokers. Those who were drinking alcohol at least one time per week for more than half a year were regarded as current alcohol drinkers^36^. Physical activity was considered as those who regularly exercised more than 20 min per day for more than 6 months^37^,^38^. Physical examination was performed on all participants at DMC-owned hospitals by trained physicians, nurses and technicians. Standing height, body weight and waist circumference were measured in individuals with light indoor clothing and without shoes. Clinical examinations in the liver, gall bladder, spleen, kidney, prostate (for males) and uterus, ovaries and fallopian tubes (for females) were conducted^35^. Body mass index (BMI) was calculated as weight (kilogram) divided by height (meter) squared. Hypertension was defined as individuals with self-reported physician diagnosis of hypertension, or blood pressure ≥140/90 mmHg, or current use of antihypertensive medication^39^. Diabetes was defined as self-reported physician diagnosis of diabetes, fasting glucose ≥7.0 mmol/L, or taking oral hypoglycemic medication or insulin^40^. Hyperlipidemia was defined as total cholesterol >5.72 mmol/L or triglycerides >1.70 mmol/L at medical examination, or a previous physician diagnosis of hyperlipidemia, or taking lipid lowering medication^41^.

Blood samples were collected after an overnight fast. Total cholesterol, triglycerides, high-density lipoprotein cholesterol (HDL-C), low-density lipoprotein cholesterol (LDL-C), mean platelet volume (MPV), fasting glucose and uric acid were determined at the DMC-owned hospital’s laboratory.

### Outcome ascertainment

The outcome of the present study was CHD incidence, including stable angina (SA), unstable angina (UA), non-fatal myocardial infarction (non-fatal MI), unspecified CHD and CHD death, between the baseline survey and October 2013. All retired employees were covered by DMC’s health-care service system and each participant had a unique medical insurance card number and ID, which made it easy to track disease incidence and mortality. CHD was identified through this medical insurance system and medical record reviews in the DMC-owned hospitals. The diagnosis of CHD was made based on well-accepted international standards by cardiologists in the DMC-owned hospitals. SA was defined as angina with no change in frequency, duration, or intensity of symptoms in 6 weeks. UA was defined as angina at rest, accelerated angina or new onset of severe angina. Non-fatal MI was diagnosed based on typical symptoms, ECG change, and cardiac enzyme values^42^. CHD death was determined by the recorded underlying cause of death on death certificates according to ICD-9: 410–414^43^ or ICD-10: I20–I25^44^.

### Statistical analysis

Baseline characteristics are presented as percentages for categorical variables and mean ± SD for continuous variables. Categorical data were analyzed with chi-square tests. Continuous variables were analyzed with t-tests. We used Cox’s proportional hazards regression model to evaluate the relation between green tea consumption and CHD incidence without any adjustment and adjusting for covariates including age, sex, BMI, education, smoking status, alcohol drinking status, physical activity, hypertension, hyperlipidemia, diabetes and family history of CHD. To examine whether the effect of green tea on CHD incidence was dependent on consumption of other beverages, we further adjusted for black tea, coffee, cola and juice. Stratified analyses by baseline characteristics were conducted with the same confounding variables being adjusted except for the stratified variable itself. Moreover, to analyze potential interactions between green tea and main characteristics, an interaction product term was included in the model. Difference of CHD-related biomarkers between baseline and follow-up was assessed with paired t-tests. The effects of green tea on 5-year changes of CHD-related biomarkers were analyzed by using general linear model. The covariates described in the Cox model and baseline values of the respective biomarkers were adjusted and additional adjustment for lipid-lowering medications and glucose-lowering medications were conducted in the analysis of the lipid profile and fast blood glucose changes respectively.

All statistical analyses were performed using SAS 9.3 (SAS institute Inc., Cary, NC). Differences were considered significant if the two-tailed p value was less than 0.05.

## Additional Information

**How to cite this article**: Tian, C. *et al.* Green tea consumption is associated with reduced incident CHD and improved CHD-related biomarkers in the Dongfeng-Tongji cohort. *Sci. Rep.* **6**, 24353; doi: 10.1038/srep24353 (2016).

## Figures and Tables

**Table 1:** Baseline characteristics of study population according to green tea consumption.

	Green tea consumption		*P*value 1
	No	Yes	
Participants (n)	12450	7021	
Male, n (%)	4382 (35.20)	4203 (59.86)	<0.001
Age (year)	62.5 ± 7.8	63.3 ± 7.4	<0.001
<50, n (%)	393 (3.16)	205 (2.92)	
50~, n (%)	4026 (32.33)	1700 (24.21)	
60~, n (%)	5674 (45.57)	3757 (53.51)	
≥70, n (%)	2357 (18.93)	1359 (19.35)	
Education (high school or beyond), n (%)	3820 (30.68)	2785 (39.67)	<0.001
Current alcohol drinker, n (%)	1971 (15.83)	2387 (34.00)	<0.001
Current smoker, n (%)	1677 (13.47)	1961 (27.93)	<0.001
Physical activity, n (yes, %)	1553 (12.47)	626 (8.92)	<0.001
BMI (kg/m^2^)	24.30 ± 3.39	24.47 ± 3.31	<0.001
Diabetes Mellitus, n (%)	2096 (16.84)	1069 (15.24)	0.003
Hyperlipidemia, n (%)	2334 (18.75)	1432 (20.40)	0.005
Hypertension, n (%)	3975 (31.93)	2268 (32.30)	0.590
Family History of CHD, n (%)	599 (4.81)	411 (5.85)	0.002
CHD-Related biomarkers^2^			
Blood lipids			
Triglycerides, mmol/L	1.44 ± 1.04	1.44 ± 1.19	0.836
Total Cholesterol, mmol/L	5.23 ± 0.96	5.18 ± 0.93	<0.001
HDL-C, mmol/L	1.46 ± 0.40	1.44 ± 0.39	0.045
LDL-C, mmol/L	3.04 ± 0.81	3.01 ± 0.78	0.007
Blood Glucose, mmol/L	6.00 ± 1.64	6.01 ± 1.71	0.746
MPV, fL	8.80 ± 1.99	8.69 ± 1.88	0.363
Uric acid, μmol/L	279.95 ± 78.50	301.12 ± 81.05	<0.001

Continuous variables were presented as mean ± SD; Dichotomous variables were presented as n (percentages).^1^ Chi-square test for categorical variables and t-test for continuous variables.^2^ Some CHD-related biomarkers have missing values; the total number for each biomarker was presented in Table 3.

**Table 2:** Association of green tea consumption with CHD incidence.

	Non-green tea consumers; cases/person (%)	Green tea consumers; cases/person (%)	Crude HR (95%CI)	Adjusted HR^1^ (95%CI)	Adjusted HR^2^ (95%CI)	*P* for interaction
Total	1377/12450 (11.06)	688/7021 (9.80)	**0.88 (0.80–0.96)**	**0.85 (0.77–0.94)**	**0.89 (0.81–0.98)**	
Age						
<60 years (n = 6324)	301/4419 (6.81)	127/1905 (6.67)	1.00 (0.81–1.23)	0.99 (0.79–1.25)	1.04 (0.84–1.30)	0.907
≥60 years (n = 13147)	1076/8031 (13.40)	561/5116 (10.97)	**0.80 (0.72**–**0.88)**	**0.82 (0.73**–**0.92)**	**0.84 (0.76**–**0.94)**	
Sex						
Male (n = 8585)	530/4382 (12.09)	438/4203 (10.42)	**0.84 (0.74**–**0.95)**	**0.84 (0.73**–**0.96)**	**0.86 (0.75**–**0.98)**	0.479
Female (n = 10886)	847/8068 (10.49)	250/2818 (8.87)	**0.83 (0.72**–**0.96)**	0.89 (0.76–1.03)	0.93 (0.80–1.07)	
BMI						
<25 g/m^2^ (n = 11854)	735/7689 (9.56)	384/4165 (9.22)	0.97 (0.86–1.10)	0.94 (0.83–1.08)	0.99 (0.87–1.13)	0.114
≥25 kg/m^2^ (n = 7617)	642/4761 (13.48)	304/2856 (10.64)	**0.76 (0.66**–**0.87)**	**0.76 (0.66**–**0.88)**	**0.79 (0.68**–**0.91)**	
Diabetes Mellitus						
No (n = 16296)	1056/10349 (10.20)	551/5947 (9.27)	0.90 (0.81–1.00)	**0.87 (0.78**–**0.98)**	0.92 (0.82–1.03)	0.337
Yes (n = 3165)	321/2096 (15.31)	137/1069 (12.82)	**0.81 (0.66**–**0.98)**	**0.78 (0.63**–**0.97)**	**0.79 (0.63**–**0.98)**	

^1^Adjusted for age, sex, BMI, education, smoking status, alcohol drinking status, physical activity, hypertension, hyperlipidemia, diabetes, family history of CHD at baseline, except for the stratified variable itself.^2^ Additionally adjusted for consumption of other beverages including black tea, coffee, cola and juice.

**Table 3:** Five years’ change in CHD-related biomarkers related to green tea consumption.

	Baseline	5-year follow-up	Non-green tea consumers	Δ	Green tea consumers				N^2^	*P* value^3^
			*P* for change^1^		Baseline	5-year follow-up	*P* for change^1^	Δ		
Blood Lipids										
Triglycerides, mmol/L	1.44 ± 1.04	1.52 ± 1.06	<0.001	0.09 ± 1.04	1.44 ± 1.19	1.47 ± 1.00	0.077	0.03 ± 1.13	15586	**0.045**^4^
Total Cholesterol, mmol/L	5.23 ± 0.96	4.85 ± 1.08	<0.001	−0.37 ± 1.01	5.18 ± 0.93	4.73 ± 1.13	<0.001	−0.43 ± 1.1	15687	**0.042**^4^
HDL-C, mmol/L	1.46 ± 0.40	1.51 ± 0.45	<0.001	0.06 ± 0.49	1.44 ± 0.39	1.51 ± 0.46	<0.001	0.07 ± 0.5	15577	**<0.001**^4^
LDL-C, mmol/L	3.04 ± 0.81	2.75 ± 0.86	<0.001	−0.29 ± 0.85	3.01 ± 0.78	2.67 ± 0.86	<0.001	−0.33 ± 0.85	15658	0.070^4^
Blood Glucose, mmol/L	6.00 ± 1.64	6.05 ± 1.71	0.028	0.03 ± 1.54	6.01 ± 1.71	6.03 ± 1.70	0.885	0 ± 1.59	15371	0.517^5^
MPV, fL	9.11 ± 1.82	8.72 ± 1.81	<0.001	−0.39 ± 2.29	9.08 ± 1.81	8.53 ± 1.77	<0.001	−0.55 ± 2.18	12306	**0.006**
Uric Acid, μmol/L	279.8 ± 78.34	308.83 ± 89.12	<0.001	29.03 ± 77.83	301.17 ± 81.09	322.61 ± 96.38	<0.001	21.44 ± 83.21	15530	**0.028**

Variables were presented as mean ± SD. ^1^Paired t-test was used to determine the change of the biomarkers over the five-year follow-up. ^2^Some CHD-related biomarkers have missing values. ^3^Generalized linear model was used to compare the difference in changes of CHD-related biomarkers over five years between green tea consumers and non-green tea consumers with adjustment for age, sex, BMI, education, smoking status, alcohol drinking status, physical activity and the baseline level of each biomarker. ^4^Additionally adjusted for lipid lowering medication. ^5^Additionally adjusted for hypoglycemic medication and insulin.
