# Corrigendum: Green tea consumption is associated with reduced incident CHD and improved CHD-related biomarkers in the Dongfeng-Tongji cohort

**DOI:** 10.1038/srep45949

**Published:** 2017-05-31

**Authors:** Chong Tian, Qiao Huang, Liangle Yang, Sébastien Légaré, Francesca Angileri, Handong Yang, Xiulou Li, Xinwen Min, Ce Zhang, Chengwei Xu, Jing Yuan, Xiaoping Miao, Mei-an He, Tangchun Wu, Xiaomin Zhang

Scientific Reports 6: Article number: 24353; 10.1038/srep24353 published online: 04 13 2016; updated: 05 31 2017.

The original version of this Article contained errors in the order of the authors’ affiliations, and an additional affiliation for Chong Tian was omitted. The affiliations for all authors are listed as below:

1 Department of Occupational and Environmental Health and Ministry of Education Key Lab for Environment and Health, School of Public Health, Tongji Medical College, Huazhong University of Science and Technology, Wuhan, China.

Chong Tian, Liangle Yang, Sébastien Légaré, Francesca Angileri, Jing Yuan, Mei-An He, Tangchun Wu, Xiaomin Zhang

2 School of Nursing, Tongji Medical College, Huazhong University of Science and Technology, Wuhan, China.

Chong Tian, Qiao Huang

3 Dongfeng Central Hospital, Dongfeng Motor Corporation and Hubei University of Medicine, Shiyan, China.

Handong Yang, Xiulou Li, Xinwen Min, Ce Zhang, Chengwei Xu

4 Department of Epidemiology and Statistics, School of Public Health, Tongji Medical College, Huazhong University of Science and Technology, 13 Hangkong Rd, Wuhan, China.

Xiaoping Miao

This has now been corrected in the HTML and PDF versions of this Article.

